# Current Evidence on Late-Onset Hypogonadism in Primary Care: A Scoping Review Protocol

**DOI:** 10.3390/jcm15207776

**Published:** 2026-10-09

**Authors:** Naohiro Tashiro, Yuki Otsuka, Yoshiaki Soejima, Yohei Masuda, Kazuki Tokumasu, Yasue Sakurada, Nobuyoshi Matsuki, Eiko Mitsuda, Koji Shiraishi, Mikiya Nakatsuka, Fumio Otsuka

**Affiliations:** 1 Department of General Medicine, Okayama University Graduate School of Medicine, Dentistry and Pharmaceutical Sciences, 2-5-1 Shikata-cho, Kita-ku, Okayama 700-8558, Japan; 2 Kato & Namiki-dori Clinic, Okayama 702-8058, Japan; 3 Department of Urology, Graduate School of Medicine, Yamaguchi University, Ube 755-8505, Japan; 4 Faculty of Health Sciences, Okayama University, Okayama 700-8558, Japan

**Keywords:** primary health care, general practice, testosterone, late-onset hypogonadism, scoping review

## Abstract

Fatigue, loss of energy, low sexual desire and erectile dysfunction are common in primary care, and late-onset hypogonadism (LOH), defined according to symptoms and low testosterone levels, is one possible explanation. Its symptoms are non-specific, and terminology and diagnostic thresholds vary between guidelines. Testosterone prescribing has risen in the UK, yet men requiring treatment may remain unidentified. Evidence has primarily been derived from specialist settings and intervention trials, and primary care evidence has not been systematically mapped. The aim of this scoping review is to map evidence on the frequency and clinical presentation of LOH in primary care; its recognition, assessment and management; and associated barriers and facilitators, and to identify evidence gaps relevant to primary care practice to inform future research. This review will follow JBI principles, and the outcomes will be reported in accordance with the Preferred Reporting Items for Systematic Reviews and Meta-Analyses extension for Scoping Reviews (PRISMA-ScR). This review will cover primary care and general practice settings internationally. PubMed, CINAHL and Web of Science Core Collection will be searched for peer-reviewed, original research published in English from 2000 onwards. Two reviewers will independently screen titles/abstracts and full texts, with disagreements resolved by discussion or a third reviewer. Data will be extracted by one reviewer and verified by another. Descriptive and basic qualitative content analyses will be conducted. This review will clarify evidence and identify knowledge gaps concerning LOH in primary care, informing future research and guidance.

## 1. Introduction

Fatigue, loss of energy, low sexual desire and erectile dysfunction are common among middle-aged and older men in primary care. These non-specific symptoms overlap with aging, obesity, type 2 diabetes, depression and sleep disorders; late-onset hypogonadism (LOH) is one possible explanation [1]. Primary care clinicians must therefore decide whether to measure testosterone, how to interpret borderline results, whether to initiate treatment and when to refer patients to specialist services [2]. Recent evidence informs these decisions, including by clarifying the cardiovascular safety of testosterone-replacement therapy in men with pre-existing cardiovascular risk and directing attention to reversing obesity-related functional hypogonadism through weight loss before testosterone therapy is considered [3,4].

These factors may lead to both over-investigation and underdiagnosis. Testosterone prescriptions increased markedly in the UK and the USA during the 2000s, with preparations dispensed in the UK increasing by almost 90% between 2001 and 2010 [5,6]. Over a comparable period in North East England, primary care requests for serum testosterone measurement rose by 137%, while the proportion of men with levels below 6.0 nmol/L changed little, and that of men with levels of 6.0–8.9 nmol/L, where diagnosis is least certain, more than doubled from 7.5% to 17.0% [5]. Conversely, an analysis of 6781 English general practices estimated that the number of men receiving treatment was only 8.1% of that expected based on age profiles, although this estimate relies on an assumed prevalence that varies with diagnostic criteria [7]. Thus, increased testing may produce borderline results while men who require treatment remain unidentified.

A further challenge is the lack of a consistent definition of LOH, which requires the presence of both low serum testosterone levels and associated symptoms [8]. Terminology and diagnostic thresholds are also inconsistent among professional bodies [1,9,10,11]. In 2018, the American Urological Association replaced *hypogonadism*, which had come to mean low testosterone production alone, with *testosterone deficiency*, which requires accompanying symptoms or signs [9], whereas the Endocrine Society retained *hypogonadism* [1]. *Andropause* was used historically [12], and the term *functional hypogonadotropic hypogonadism*, in which organic disease is excluded, has recently been proposed [13]. Diagnostic testosterone thresholds also differ between guidelines [14].

The criteria applied therefore determine whether an individual is considered to have LOH. In the European Male Ageing Study (EMAS), 17.0% of community-dwelling men had total testosterone levels below 11 nmol/L, whereas only 2.1% met the diagnostic criteria for LOH, which also required three sexual symptoms and low free testosterone [8]. In a US primary care study of men aged ≥45 years, 38.7% met a biochemical definition without symptom requirements [15], where the threshold of <300 ng/dL (approximately 10.4 nmol/L) was similar to that used in the EMAS. These contrasting estimates illustrate how prevalence varies with population and diagnostic criteria.

Most available evidence derives from specialist settings or trials evaluating testosterone replacement therapy. Existing guidelines have been produced by specialist societies and are not intended for use in primary care [11]. Primary care is the first point of contact for most men, yet evidence on the frequency, presentation, recognition, assessment and management of LOH in this setting has not been systematically mapped. Barriers and facilitators at each stage are also unclear. Men’s reluctance to seek help from health professionals is well documented [16,17] and may further contribute to delayed presentation and under-recognition of LOH in primary care.

Given the heterogeneity in terminology, diagnostic criteria, study design and setting, a scoping review is appropriate to map the evidence and identify knowledge gaps. On 24 June 2026, a preliminary search of Open Science Framework (OSF) Registries, JBI Evidence Synthesis and PubMed (MEDLINE), using terms including *hypogonadism*, *testosterone deficiency*, *andropause*, *low testosterone* and *testosterone replacement therapy*, identified no current or ongoing scoping review of LOH in primary care. PROSPERO, which does not accept scoping reviews for registration, was also searched, and no systematic review on this topic was identified. Therefore, this review is intended to map the frequency and clinical presentation of LOH in primary care; its recognition, assessment and management; associated barriers and facilitators; and evidence gaps for practice and future research.

## 2. Materials and Methods

This scoping review will be conducted using the JBI methodology [18], and this protocol was developed according to best practice guidance for scoping review protocols [19]. The review will be reported in accordance with the Preferred Reporting Items for Systematic Reviews and Meta-Analyses extension for Scoping Reviews (PRISMA-ScR) [20]. This protocol was developed with reference to the PRISMA-ScR checklist, which was adapted for the protocol stage (Supplementary Table S1). Any protocol deviations will be reported and explained in the subsequent review. Scoping reviews are ineligible for registration in PROSPERO; however, JBI guidance recognizes registration with OSF or Figshare or the publication of the protocol in a journal as means of making it publicly available [18]. We have therefore chosen to make this protocol publicly available by publishing it in a journal and have not registered it with OSF.

### 2.1. Objectives

This review will map LOH in primary care from both patient and clinician perspectives, guided by the following five sub-questions:

1. How frequently is LOH reported among men attending primary care, and what are the characteristics of the affected men?
2. What symptoms, presenting complaints and reasons for consultation are described for men with suspected or confirmed LOH?
3. How is LOH recognized in primary care, including its consideration as a differential diagnosis and delayed or missed diagnosis, and what barriers and facilitators influence recognition?
4. How is LOH screened for and assessed, and what barriers and facilitators are associated with screening and assessment?
5. How is LOH managed, including referral to or shared care with specialist services, and what barriers and facilitators are associated with this?

### 2.2. Eligibility Criteria

Eligibility criteria were developed using the Population, Concept and Context (PCC) framework according to JBI guidance [18].

#### 2.2.1. Population

Eligible sources will concern adult men (≥18 years) with LOH or age-related or functional testosterone deficiency. This threshold was chosen because lower age limits differ across studies (e.g., 40 years in the EMAS [8] and 45 years in the US primary care study [15]) and because primary care sources often report mixed-age cohorts without an age-stratified LOH subgroup, so narrowing the range a priori risks excluding relevant data. Age will be extracted for all included sources (Supplementary Box S1, item 8) to allow age-related patterns to be described in the review. Primary care clinicians, including physicians and nurse practitioners, will also be considered where clinician recognition or practice is examined. Sources limited to organic, congenital, drug-induced or iatrogenic causes; women or children; and animal or laboratory studies will be excluded. Mixed populations will be included where relevant data can be extracted separately. Sources addressing low testosterone without a specified etiology will be included by default. Where data on men without organic, congenital, drug-induced or iatrogenic causes cannot be extracted separately, mixed-etiology cohorts will be excluded if men with these causes constitute more than half of the sample and included if this proportion is not reported. The etiological composition of each cohort will be extracted where reported. Given the variation in terminology and diagnostic criteria, a broad concept of LOH will be used, and each source’s definition of LOH will be extracted.

#### 2.2.2. Concept

Eligible sources will address any of the five sub-questions, including associated barriers and facilitators. For sub-questions 3 and 4, recognition refers to whether and how LOH is considered a possible explanation for a man’s presentation up to the point at which the decision to investigate is made, whereas assessment refers to the investigations and evaluation undertaken once LOH is considered. Data that cannot be separated will be charted under both sub-questions and flagged. Trials primarily evaluating the efficacy or safety of testosterone replacement therapy will be excluded, even when conducted in primary care, but sources describing such therapy as part of primary care management will be included. For example, a randomized trial in general practice comparing testosterone with placebo on sexual function would be excluded, whereas a study evaluating how men prescribed testosterone are monitored in general practices, or a trial of a primary care pathway for initiating and monitoring treatment, would be included.

#### 2.2.3. Context

Eligible settings will provide first-contact care accessible without referral, including primary care, general practice, family medicine, general medicine and general internal medicine, in any country or health system. Studies of primary care attenders or records will be eligible. General population studies will be excluded unless primary care is explicitly identified as the context. Specialist settings (urology, endocrinology or andrology) will be included only where a specific link with primary care is reported, such as patients referred from primary care, referral criteria or shared care. Hospital-based general medicine or general internal medicine departments will be eligible if the source reports that they provide first-contact, walk-in or non-referral-based care. Departments that primarily serve referred patients will be excluded, even if designated as general medicine or general internal medicine.

#### 2.2.4. Types of Sources

Original quantitative, qualitative and mixed-methods studies published in peer-reviewed journals will be eligible. Reviews, commentaries, editorials, case reports or series, guidelines, consensus statements and patient education materials will be excluded because this review will focus on primary research relevant to patterns of primary care practice rather than individual cases, recommendations or secondary evidence. Gray literature, including conference abstracts and proceedings, preprints and theses, will also be excluded so that the evidence base comprises studies that have undergone journal peer review. Conference abstracts rarely report LOH definitions, testosterone measurement and the sampling required for charting in detail, and findings from preprints and theses are often subsequently published in peer-reviewed journals, where they can be captured in the database searches. Although guidelines will not be included as sources, the guidelines followed in included sources will be extracted (Supplementary Box S1, item 20), allowing their role in shaping reported practice to be described. In the discussion section of the review, the findings will also be compared with the recommendations of key guidelines [1,9,14] to identify where primary care practice aligns with or diverges from them.

### 2.3. Search Strategy

The three-step JBI search strategy will be applied [18]. First, an initial PubMed (MEDLINE) and CINAHL search was performed by NT to identify relevant text words and index terms, which informed full strategies combining controlled vocabulary and free-text terms adapted for each database. As a Peer Review of Electronic Search Strategies (PRESS) by an information specialist was unavailable, all co-authors other than NT reviewed the strategies before finalization.

Second, after this protocol is accepted for publication, PubMed (MEDLINE), CINAHL (EBSCO) and Web of Science (WoS) Core Collection will be searched. The WoS Core Collection editions to be searched are listed in Supplementary Section S1; Embase and Scopus are unavailable. English-language sources published from 2000 onwards will be considered because the aim of this review is to map the international evidence base and because the contemporary concept of LOH and the associated clinical guidelines were established within this period. Including the Japanese database Ichushi-Web was considered, but a preliminary check identified few potentially eligible original Japanese-language articles. Given the review’s focus on internationally comparable concepts rather than locally specific constructs, Ichushi-Web will not be searched. Full strategies are provided in Supplementary Section S1.

In the third step, the reference lists of all included full-text sources will be screened for additional eligible studies.

### 2.4. Studies and Source of Evidence Selection

Citations will be deduplicated in EndNote 2025 (Clarivate, Philadelphia, PA, USA). NT and YSo will independently perform pilot screening on 50 randomly selected titles and abstracts [18,20]. Agreement will be assessed using both raw percent agreement and Cohen’s kappa (*κ*). If agreement is <75% or *κ* is <0.60 [21], criteria will be clarified and the pilot repeated on a further 50 randomly selected records. The same applies if too few records are judged potentially eligible for *κ* to be meaningfully estimated. They will then independently screen titles/abstracts and full texts, with disagreements resolved by discussion or with input from YO. Reasons for full-text exclusion will be recorded. Multiple reports of the same study population will be linked to avoid double counting. Selection will be reported in a PRISMA-ScR flow diagram.

### 2.5. Data Extraction

Data will be extracted using an instrument developed for this review (Supplementary Box S1). The instrument will cover source characteristics, population and setting, including the sampling frame; LOH definition and testosterone measurement methodology; patient characteristics; clinician practices, including management and referral; and barriers and facilitators. NT and YSo will pilot the instrument on two or three sources of each type and refine it as necessary. NT will then extract all data; YSo will verify all entries, and disagreements will be adjudicated by YO. Any subsequent modifications to the instrument will be reported.

### 2.6. Critical Appraisal

Critical appraisal will not be undertaken because the review is intended to map rather than evaluate the available evidence.

### 2.7. Data Analysis and Presentation

Data will be summarized descriptively using frequency counts, tables and evidence maps aligned with the five sub-questions. Frequency and clinical presentation findings will be grouped and reported according to the LOH definition and diagnostic criteria applied in each source (Supplementary Box S1, items 12–13) to allow for explicit comparison across differing definitional approaches. The findings will be presented separately by setting category (primary care/general practice/family medicine, general medicine/general internal medicine and specialist settings linked to primary care) where the data allow, given differences in first-contact function across health systems. Data on barriers and facilitators will undergo basic qualitative content analysis following JBI guidance [22]. The data will first be allocated deductively to three levels (patient, clinician and health system), within which codes and categories will be developed inductively. NT will code all sources, and the codes and categories will be reviewed by all other authors, with disagreements resolved through discussion. In line with JBI guidance, this analysis will remain descriptive and will not proceed to abstraction or reinterpretation. Interpretive approaches, such as thematic synthesis or meta-aggregation, and meta-analysis will not be undertaken. A narrative summary will accompany the tables and evidence maps.

### 2.8. Timeline

In total, screening, data extraction and analysis are expected to take approximately two months from the start of searching the databases, with completion of the review anticipated shortly thereafter.

## 3. Discussion

### 3.1. Summary

To our knowledge, this will be the first scoping review to map the frequency, clinical presentation, recognition, assessment and management of LOH in primary care, including its associated barriers and facilitators.

### 3.2. Strengths and Limitations

The strengths of this review include its adherence to established scoping review methodology and reporting standards, independent screening by two reviewers, and five sub-questions addressing both patient characteristics and clinical practice. However, this review has several limitations. First, the search will be restricted to three databases because Embase and Scopus are unavailable, although reference list screening may identify additional sources. Second, gray literature, including conference abstracts and preprints, will be excluded, so recent findings not yet published in peer-reviewed journals will not be captured. Third, the restriction to English-language sources means that relevant sources published in other languages, including the Japanese-language primary care literature on LOH indexed in Ichushi-Web, will not be captured. However, a preliminary check of Ichushi-Web identified few potentially eligible original articles. Fourth, no critical appraisal will be undertaken, so the certainty of the evidence will not be assessed. Fifth, there will be no patient or public involvement, which would be valuable given the review’s focus on help-seeking.

### 3.3. Comparison with the Existing Literature

The reviews of LOH published to date offer practical guidance for primary care clinicians based on specialist recommendations [23] or compare the diagnostic and management recommendations of specialist society guidelines [24] without systematically reviewing primary studies. In contrast, this review will map primary evidence on the presentation, recognition, assessment and management of LOH in primary care.

### 3.4. Implications for Research and Practice

This review will clarify how LOH definitions, diagnostic thresholds and assessment methods are applied in primary care and how variation may affect clinical practice. Identified gaps should inform future primary care research and the development of guidance for this setting.

## Data Availability

No new data will be generated for this protocol. All sources for the subsequent review will be drawn from the published literature, and the full search strategies and the data extraction instrument are provided in the Supplementary Materials.

## Supplementary Materials

The following supporting information can be downloaded at: https://www.mdpi.com/article/10.3390/jcm15207776/s1, Section S1: Search strategies for all databases. Box S1: Data extraction instrument. Table S1: PRISMA-ScR checklist adapted for the protocol stage.
