# Supplementary material for: Current Evidence on Late-Onset Hypogonadism in Primary Care: A Scoping Review Protocol
Source: J Clin Med. 2026 Oct 9;15(20):7776. doi: 10.3390/jcm15207776 (PMC13705377; doi:10.3390/jcm15207776)
Supplement: Supplementary file 1 [file jcm-15-07776-s001.zip › jcm-4592644-supplementary.pdf]

Supplementary data

Section S1. Search strategies

The search strategy was structured around the Concept and Context elements of the PCC framework:

Concept: late-onset hypogonadism

Context: primary care settings

No separate search block was constructed for the population element, as late-onset hypogonadism is by definition a condition of men; restricting the search by sex would have reduced sensitivity without improving precision.

S1.1 MEDLINE (via PubMed)

Interface	PubMed (https://pubmed.ncbi.nlm.nih.gov/)
Limits	2000 onwards; English (applied using the interface filters)

#1 Concept block

```
("Hypogonadism"[Mesh] OR hypogonad*[tiab] OR LOH[tiab] OR "Testosterone/deficiency"[Mesh] OR "testosterone deficien*"[tiab] OR "androgen deficien*"[tiab] OR "Andropause"[Mesh] OR andropaus*[tiab] OR "male menopaus*"[tiab] OR "male climacteric*"[tiab] OR "ageing male syndrome*"[tiab] OR "aging male syndrome*"[tiab] OR "low testosterone"[tiab] OR "testosterone replacement therap*"[tiab] OR "testosterone prescri*"[tiab])
```

#2 Context block

```
("Primary Health Care"[Mesh] OR "General Practice"[Mesh] OR "General Practitioners"[Mesh] OR "Physicians, Family"[Mesh] OR "Physicians, Primary Care"[Mesh] OR "primary care"[tiab] OR "primary health care"[tiab] OR "primary healthcare"[tiab] OR "general practice*"[tiab] OR "general practitioner*"[tiab] OR "general physician*"[tiab] OR generalist*[tiab] OR "family medicine"[tiab] OR "family practice*"[tiab] OR "family physician*"[tiab] OR "family doctor*"[tiab] OR internist*[tiab] OR "general internal medicine"[tiab] OR "nurse practitioner*"[tiab] OR "Nurse Practitioners"[Mesh])
```

#3 Combined set

```
#1 AND #2
```

S1.2 CINAHL (via EBSCOhost)

Interface	EBSCOhost (https://research.ebsco.com/)
Limits	2000 onwards; English (applied using the interface filters)

#1 Concept block

```
( (MH "Hypogonadism") OR TI hypogonad* OR AB hypogonad* OR TI LOH OR AB LOH OR TI "testosterone deficien*" OR AB "testosterone deficien*" OR TI "androgen deficien*" OR AB "androgen deficien*" OR (MH "Andropause") OR TI andropaus* OR AB andropaus* OR TI "male menopaus*" OR AB "male menopaus*" OR TI "male climacteric*" OR AB "male climacteric*" OR TI "ageing male syndrome*" OR AB "ageing male syndrome*" OR TI "aging male syndrome*" OR AB "aging male syndrome*" OR TI "low testosterone" OR AB "low testosterone" OR (MH "Testosterone Replacement Therapy") OR TI "testosterone replacement therap*" OR AB "testosterone replacement therap*" OR TI "testosterone prescri*" OR AB "testosterone prescri*")
```

#2 Context block

```
( (MH "Primary Health Care") OR (MH "Family Practice") OR (MH "Physicians, Family") OR (MH "Nurse Practitioners") OR TI "primary care" OR AB "primary care" OR TI "primary health care" OR AB "primary health care" OR TI "primary healthcare" OR AB "primary healthcare" OR TI "general practice*" OR AB "general practice*" OR TI "general practitioner*" OR AB "general practitioner*" OR TI "general physician*" OR AB "general physician*" OR TI generalist* OR AB generalist* OR TI "family medicine" OR AB "family medicine" OR TI "family practice*" OR AB "family practice*" OR TI "family physician*" OR AB "family physician*" OR TI "family doctor*" OR AB "family doctor*" OR TI internist* OR AB internist* OR TI "general internal medicine" OR AB "general internal medicine" OR TI "nurse practitioner*" OR AB "nurse practitioner*")
```

#3 Combined set

```
#1 AND #2
```

S1.3 Web of Science Core Collection (via Clarivate)

Interface	Web of Science, Advanced Search (https://www.webofscience.com/)
Editions searched	SCI-EXPANDED, SSCI, A&HCI, CPCI, ESCI
Limits	2000 onwards; English (applied using the interface filters)

#1 Concept block

```
TS=(hypogonad* OR LOH OR "testosterone deficien*" OR "androgen deficien*" OR andropaus* OR "male menopaus*" OR "male climacteric*" OR "aging male syndrome*" OR "ageing male syndrome*" OR "low testosterone" OR "testosterone replacement therap*" OR "testosterone prescri*")
```

#2 Context block

```
TS=("primary care" OR "primary health care" OR "primary healthcare" OR "general practice*" OR "general practitioner*" OR "general physician*" OR generalist* OR "family medicine" OR "family practice*" OR "family physician*" OR "family doctor*" OR internist* OR "general internal medicine" OR "nurse practitioner*")
```

#3 Combined set

```
#1 AND #2
```

Box S1. Data extraction instrument

Draft data extraction instrument. As described in the main text, the instrument will be piloted on two or three sources of each source type and finalized following team discussion before full extraction begins.

**Bibliographic details**		
1	Author(s) and year of publication	
2	Country	
3	Source (journal, volume, pages) and DOI or PMID	
**Nature of the source**		
4	Type of source	
5	Study design	
6	Aim of the study	
**Population and setting**		
7	Population studied, including etiological composition where reported	
8	Sample size and age	
9	Setting	
10	Sampling frame and recruitment method	
11	Denominator definition, where frequency or detection rate is reported	
**Definition and measurement of LOH**		
12	Terminology used	
13	Definition and diagnostic criteria	
14	Diagnostic threshold and testosterone measure(s) reported (total, free, bioavailable or calculated free)	
15	Assay platform (immunoassay vs LC–MS/MS), if reported	
16	Timing of sampling (morning vs opportunistic), if reported	
17	Single or repeated confirmatory measurement	

18	Ancillary hormones measured (SHBG, LH, FSH), if reported	
19	Symptom instrument used (e.g. ADAM, AMS, IIEF or ad hoc), if reported	
20	Guideline followed, if any	
**Sub-question 1 - frequency of LOH**		
21	Frequency as reported	
22	Characteristics of the men affected	
**Sub-question 2 - clinical presentation**		
23	Symptom profile, presenting complaints and reasons for consultation	
**Sub-question 3 - recognition**		
24	Clinician recognition of LOH, including delayed or missed diagnosis	
**Sub-question 4 - screening and assessment**		
25	Screening and assessment undertaken, including tools used	
**Sub-question 5 - management**		
26	Management, monitoring and referral to specialist services	
**Barriers and facilitators**		
27	Barriers and facilitators reported, with the corresponding sub-question indicated	
**Other**		
28	Key findings	
29	Limitations reported by the authors and reviewer notes	

Table S1. PRISMA-ScR checklist adapted for the protocol stage

The checklist item wording (columns 1-3) is reproduced verbatim from the PRISMA-ScR checklist; the final column indicates where each item is addressed in this protocol, or notes items not yet applicable at the protocol stage.

Item	#	PRISMA-ScR checklist item	Location in this protocol
**TITLE**			
Title	1	Identify the report as a scoping review.	Title
**ABSTRACT**			
Structured summary	2	Provide a structured summary that includes (as applicable): background, objectives, eligibility criteria, sources of evidence, charting methods, results, and conclusions that relate to the review questions and objectives.	Abstract
**INTRODUCTION**			
Rationale	3	Describe the rationale for the review in the context of what is already known. Explain why the review questions/objectives lend themselves to a scoping review approach.	Introduction
Objectives	4	Provide an explicit statement of the questions and objectives being addressed with reference to their key elements (e.g., population or participants, concepts, and context) or other relevant key elements used to conceptualize the review questions and/or objectives.	Introduction, sixth paragraph; Objectives; Eligibility criteria
**METHODS**			
Protocol and registration	5	Indicate whether a review protocol exists; state if and where it can be accessed (e.g., a Web address); and if available, provide registration information, including the registration number.	Material and Method, first paragraph

Item	#	PRISMA-ScR checklist item	Location in this protocol
Eligibility criteria	6	Specify characteristics of the sources of evidence used as eligibility criteria (e.g., years considered, language, and publication status), and provide a rationale.	Eligibility criteria; Search strategy, second paragraph
Information sources	7	Describe all information sources in the search (e.g., databases with dates of coverage and contact with authors to identify additional sources), as well as the date the most recent search was executed.	Search strategy; Supplementary Section S1
Search	8	Present the full electronic search strategy for at least 1 database, including any limits used, such that it could be repeated.	Supplementary Section S1
Selection of sources of evidence†	9	State the process for selecting sources of evidence (i.e., screening and eligibility) included in the scoping review.	Study and source of evidence selection
Data charting process‡	10	Describe the methods of charting data from the included sources of evidence (e.g., calibrated forms or forms that have been tested by the team before their use, and whether data charting was done independently or in duplicate) and any processes for obtaining and confirming data from investigators.	Data extraction
Data items	11	List and define all variables for which data were sought and any assumptions and simplifications made.	Data extraction; Supplementary Box S1
Critical appraisal of individual sources of evidence§	12	If done, provide a rationale for conducting a critical appraisal of included sources of evidence; describe the methods used and how this information was used in any data synthesis (if appropriate).	Critical appraisal
Synthesis of results	13	Describe the methods of handling and summarizing the data that were charted.	Data analysis and presentation
**RESULTS**			

Item	#	PRISMA-ScR checklist item	Location in this protocol
Selection of sources of evidence	14	Give numbers of sources of evidence screened, assessed for eligibility, and included in the review, with reasons for exclusions at each stage, ideally using a flow diagram.	Planned as a PRISMA-ScR flow diagram (Study and source of evidence selection); Not yet applicable at the protocol stage; to be reported in the completed review
Characteristics of sources of evidence	15	For each source of evidence, present characteristics for which data were charted and provide the citations.	Planned (Data analysis and presentation); Not yet applicable at the protocol stage; to be reported in the completed review
Critical appraisal within sources of evidence	16	If done, present data on critical appraisal of included sources of evidence (see item 12).	Not applicable (critical appraisal will not be undertaken)
Results of individual sources of evidence	17	For each included source of evidence, present the relevant data that were charted that relate to the review questions and objectives.	Planned (Data analysis and presentation); Not yet applicable at the protocol stage; to be reported in the completed review
Synthesis of results	18	Summarize and/or present the charting results as they relate to the review questions and objectives.	Planned (Data analysis and presentation); Not yet applicable at the protocol stage; to be reported in the completed review
**DISCUSSION**			

Item	#	PRISMA-ScR checklist item	Location in this protocol
Summary of evidence	19	Summarize the main results (including an overview of concepts, themes, and types of evidence available), link to the review questions and objectives, and consider the relevance to key groups.	Not yet applicable at the protocol stage; to be reported in the completed review
Limitations	20	Discuss the limitations of the scoping review process.	Discussion, Strengths and limitations (anticipated limitations)
Conclusions	21	Provide a general interpretation of the results with respect to the review questions and objectives, as well as potential implications and/or next steps.	Discussion, Implications for research and practice
**FUNDING**			
Funding	22	Describe sources of funding for the included sources of evidence, as well as sources of funding for the scoping review. Describe the role of the funders of the scoping review.	Funding

JBI = Joanna Briggs Institute; PRISMA-ScR = Preferred Reporting Items for Systematic reviews and Meta-Analyses extension for Scoping Reviews.

Where sources of evidence (see second footnote) are compiled from, such as bibliographic databases, social media platforms, and Web sites.

† A more inclusive/heterogeneous term used to account for the different types of evidence or data sources (e.g., quantitative and/or qualitative research, expert opinion, and policy documents) that may be eligible in a scoping review as opposed to only studies. This is not to be confused with information sources (see first footnote).

‡ The frameworks by Arksey and O'Malley and Levac and colleagues and the JBI guidance refer to the process of data extraction in a scoping review as data charting.

§ The process of systematically examining research evidence to assess its validity, results, and relevance before using it to inform a decision. This term is used for items 12 and 19 instead of "risk of bias" (which is more applicable to systematic reviews of interventions) to include and acknowledge the various sources of evidence that may be used in a scoping

review (e.g., quantitative and/or qualitative research, expert opinion, and policy document).

Checklist reproduced from: Tricco AC, Lillie E, Zarin W, et al. PRISMA Extension for Scoping Reviews (PRISMA-ScR): Checklist and Explanation. Ann Intern Med 2018; 169(7): 467–473. DOI: https://doi.org/10.7326/M18-0850
